# Case report: Effect of Hb E heterozygosity on HbA1c value by the Tosoh HLC-723G11

**DOI:** 10.3389/fpubh.2023.1217662

**Published:** 2023-08-03

**Authors:** Wei Gao, Wenyu Li, Qian Zheng, Nenggang Jiang, Huairong Tang

**Affiliations:** ^1^Health Management Center, General Practice Center, West China Hospital, Sichuan University, Chengdu, China; ^2^West China School of Medicine, Sichuan University, Chengdu, China; ^3^Department of Laboratory Medicine, West China Hospital, Sichuan University, Chengdu, China

**Keywords:** HbA1c, Hb E, heterozygosity, high-pressure liquid chromatography, Tosoh HLC-723G11

## Abstract

**Objective:**

We report the effect of Hb E heterozygosity on HbA1c value by the Tosoh HLC-723G11.

**Case report:**

A 45 years-old Chinese woman presented with an abnormally low HbA1c level of 3.7% (3.9%–6.1%) in a health examination. Fasting blood glucose was normal. Blood routine examination and serum bilirubin were in the normal range. HbA1c was determined by Tosoh HLC-723G11. There was an abnormal peak between A1c and A0 on the chromatogram. Hemoglobin electrophoresis indicated that the Hb E zone accounted for 25.1%. The β-thalassemia-related genes (mutant type) were βE M/N, and the related gene CD26 (A > G) was mutated. OGTT indicated prediabetes.

**Conclusion:**

Hb E heterozygosity may reduce HbA1c value with abnormal chromatograms, as determined by a Tosoh HLC G11 analyzer. The Tosoh HLC G11 analyzer can well identify Hb E variation. In this case, further blood glucose-related tests should be performed to avoid missed diagnoses. However, a large sample size is needed to confirm this conclusion.

## 1. Introduction

Glycosylated hemoglobin A1c (HbA1c) is an important method for monitoring blood glucose and diagnosing diabetes mellitus (1–3). At present, the popular methods for detecting HbA1c include ion exchange high-performance liquid chromatography (HPLC), boric acid affinity chromatography, capillary electrophoresis, immunoassays and enzyme assays (4, 5). Ion exchange HPLC is widely used in clinical practice, but the HbA1c values obtained by ion exchange HPLC are susceptible to hemoglobin variants (6). The same hemoglobin variation has different effects on HbA1c detection by different ion exchange HPLC analyzers. Hb E is the most common variant in southern China (7). Here, we report a case of abnormally low HbA1c with an abnormal chromatogram by the Tosoh HLC-723G11 caused by Hb E heterozygosity.

## 2. Case presentation

A 45 years-old Chinese woman presented with an abnormally low HbA1c level of 3.7% (3.9%–6.1%) in a health examination. The fasting blood glucose level was 105.9 mg/dL (normal range 70.2–106.2). She had a history of hypertension. There was no other medical history or complaints. Her father suffered from diabetes and received treatment. No positive signs were found in the physical examination. Her body mass index was 27.45 kg/m^2^. Blood routine examination and serum bilirubin were in the normal range. The details of the laboratory examination are shown in Table 1.

**Table 1:** Laboratory results of the patient.

Factors	Results	Reference range
HbA1c (%)	3.7	3.9–6.1
Fasting blood glucose (mg/dl)	105.9	70.2–106.2
Red blood cell count (*10^12^/L)	4.6	3.8–5.1
Hb (g/L)	126	115–150
MCV (fL)	97	82–100
MCH (pg)	30	27–34
MCHC (g/L)	337	316–354
Total bilirubin (μmol/L)	15.8	5.5–28.8
Direct bilirubin (μmol/L)	6.6	<8.8
Indirect bilirubin (μmol/L)	9.2	<20
Fasting plasma glucose in OGTT (mg/dL)	105.1	70.2–106.2
2 h plasma glucose (mg/dL)	157.8	59.4–140.4
Fasting insulin (uU/mL)	19.1	1.5–15.0
2 h insulin (uU/mL)	88.7	3.0–60.0
GA (%)	14.68	9–14
Fasting plasma glucose in OGTT 3 months later (mg/dL)	99.8	70.2–106.2
2 h plasma glucose 3 months later (mg/dL)	141.2	59.4–140.4
Fasting insulin 3 months later (uU/mL)	15.9	1.5–15.0
2 h insulin 3 months later (uU/mL)	72.5	3.0–60.0
GA3months later (%)	14.06	9–14

**Figure 1:**
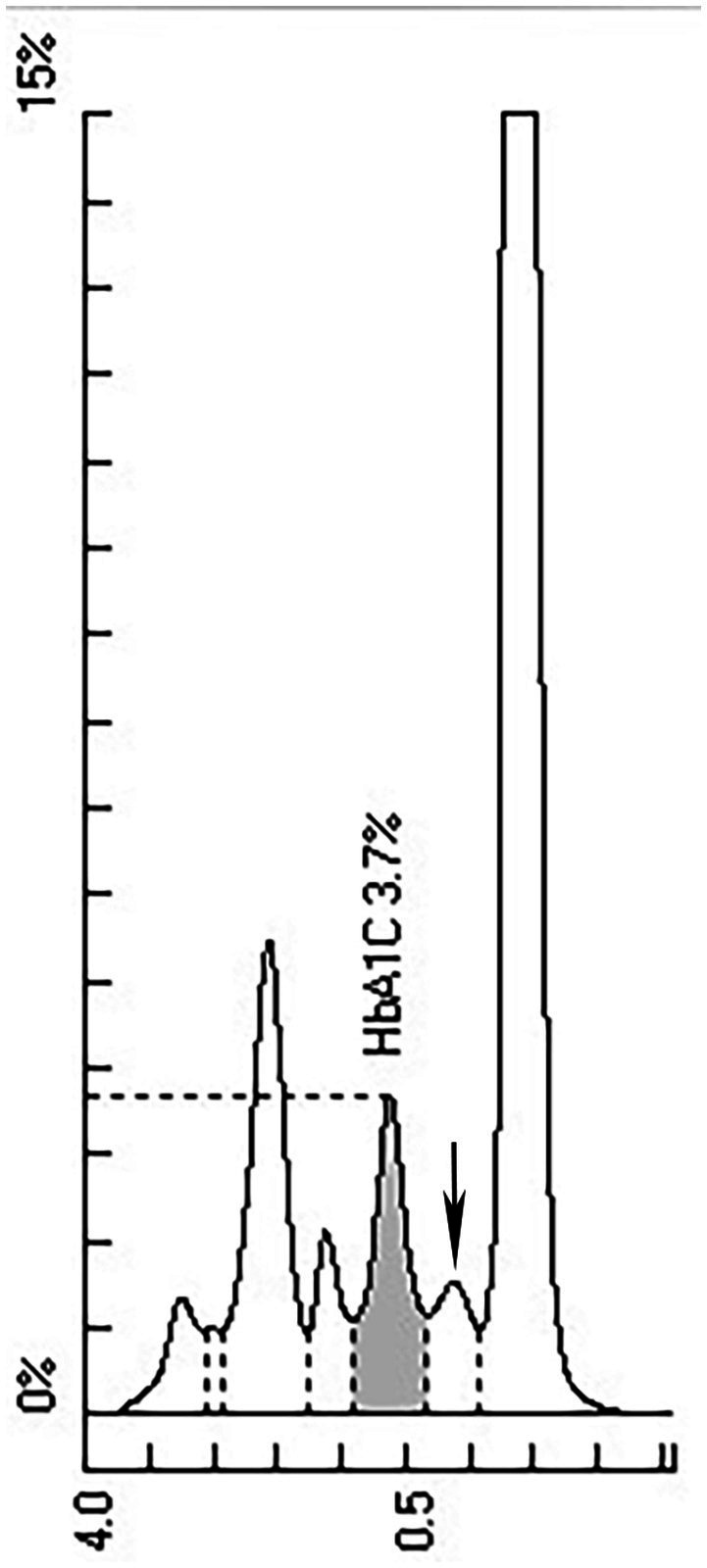
Chromatogram of HbA1c by Tosoh HLC G11 analyzer. Abnormal peak marked by arrow.

**Figure 2:**
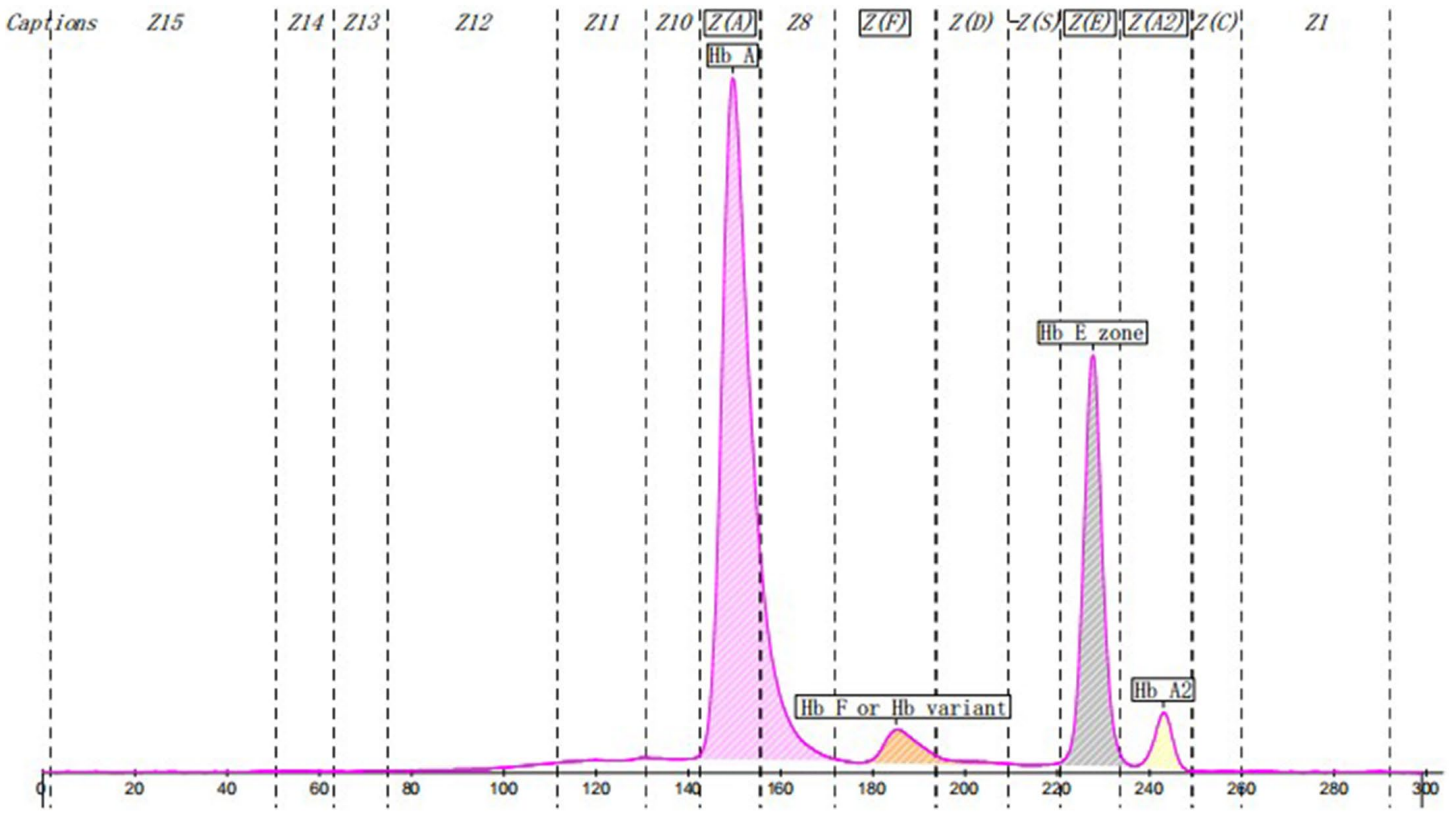
Chromatography of hemoglobin electrophoresis.

HbA1c was determined by ion exchange HPLC (Tosoh HLC-723G11, Tosoh Corporation, Shunan, Yamaguchi, Japan). There was an abnormal peak between A1c and A0 on the chromatogram (Figure 1). With the patient’s informed consent, she underwent further examination. Hemoglobin electrophoresis indicated that Hb A accounted for 67.4% (normal range 96–97.6), Hb F or Hb variant accounted for 4.3% (normal range <0.9) and Hb E zone accounted for 25.1% (normal range ≤0) (Figure 2). The β-thalassemia-related genes (mutant type) were βE M/N, and the related gene CD26 (A > G) was mutated. Glycated albumin (GA) was 14.68% (normal range 9%–14%), and the results of the OGTT and insulin release test are shown in Table 1. She was diagnosed with prediabetes. After communication with patients, the patient was given lifestyle interventions, including a controlled diet and moderate intensity aerobic exercise. Three months later, both blood glucose and hyperinsulinemia improved, and GA was 14.06%.

## 3. Discussion

Ion exchange HPLC systems commonly used in clinical practice include Bio-Rad, Tosoh and Arkray. A chromatography figure for each sample by ion exchange HPLC can help to interpret HbA1c (8). However, the HbA1c values determined by ion exchange HPLC are susceptible to hemoglobin variants (6).

Hb E is a common β-chain variant. Lysine is replaced by glutamine at codon 26 in the β globin chain. The influence of Hb E on the HbA1c value depends on the Hb E concentration and the analyzers. Previous studies found that the HbA1c value could not be measured or significantly increased or decreased by different analyzers when Hb E homozygotes were present (9–11). Hb E heterozygotes had elevated HbA1c values with abnormal chromatograms by Bio-Rad Variant or Bio-Rad Variant II Turbo analysers (11). However, other studies have found that HbA1c values were not significantly affected by Hb E heterozygotes by Variant II Turbo analysers (12). HbA1c values were lowered with abnormal chromatograms by Arkray HA-8160 and HA-8180V when Hb E heterozygotes were present (12, 13).

The widely used Tosho HLC includes Tosoh A1c 2.2 Plus, Tosoh G7 and Tosoh G8. One study found that Hb E heterozygosity slightly increased the HbA1c value by Tosoh HLC G8 (10). Some studies have reported an overall decrease in HbA1c by Tosoh A1c 2.2 Plus, Tosoh G7, and Tosoh G8 (14, 15). Other studies reported a decrease in HbA1c without an abnormal peak on the chromatogram by Tosoh G8 (12). Tosoh HLC G11 is an upgraded version of G8 and shows good accuracy and linearity in HbA1c measurement (16). However, there are few reports about the effect of Hb E on HbA1c by the Tosoh HLC G11. This study found that the HbA1c value decreased significantly with an abnormal peak between A1c and A0. The principle of ion exchange HPLC is to separate HbA1c according to the charge difference. The change in amino acids in Hb E causes the charge to change. Hb E is separated from HbA1c, which lowers the HbA1c value.

Tosoh HLC G11 has standard mode and variant mode. A study found that HbA1c results by Tosoh HLC G11 variant mode were similar to capillary electrophoresis results for samples with Hb variants (not Hb E) (16). HbA1c in our study was measured in standard mode and should also be analyzed in variant mode if conditions are available. At present, few studies have reported the effects of the G11 variant mode on HbA1c and Hb variants, so this is one of the future research points. HPLC is susceptible to the interference of Hb variants. In addition to HPLC, common methods for HbA1c include enzyme methods, immunoassays, boronate affinity and capillary electrophoresis. Due to the different detection principles, other methods do not interfere with Hb E heterozygotes (14). If the laboratory does not have these methods, GA and OGTT are also alternative methods (17). If variant hemoglobin is found, further hemoglobin electrophoresis should be recommended for differential diagnosis.

Abnormal peaks produced by Tosoh HLC G11 can help interpret HbA1c and identify Hb E variants. The HbA1c value is underestimated if Hb E heterozygous variants are present. HbA1c can be accurately measured by capillary electrophoresis, immunoassays and enzyme assays (15). This patient had a family history of diabetes and was overweight. Further examination revealed prediabetes. In this case, further blood glucose-related tests should be performed to avoid missed diagnoses after full communication with the patient. After early intervention, blood glucose and hyperinsulinemia can be improved.

## 4. Conclusion

Hb E heterozygosity may reduce the HbA1c value with an abnormal chromatogram by a Tosoh HLC G11 analyzer. The Tosoh HLC G11 analyzer can well identify Hb E variation. In this case, further blood glucose-related tests should be performed to avoid missed diagnoses. However, a large sample size is needed to confirm this conclusion.

## Data availability statement

The original contributions presented in the study are included in the article/supplementary material, further inquiries can be directed to the corresponding author.

## Ethics statement

The studies involving human participants were reviewed and approved by Ethics Committee of West China Hospital, Sichuan University. The ethics committee waived the requirement of written informed consent for participation.

## Author contributions

WG and WL contributed equally to this paper and wrote the manuscript. QZ reviewed the manuscript. NJ interpreted the laboratory results. HT was responsible for the study design and manuscript revision. All authors contributed to the article and approved the submitted version.

## Funding

This study was supported by the Science and Technology Bureau of Sichuan Province (grant numbers: 2020YFS0099 and 2019YFS0306). This paper is supported by the national clinical key specialty construction project.

## Conflict of interest

The authors declare that the research was conducted in the absence of any commercial or financial relationships that could be construed as a potential conflict of interest.

## Publisher’s note

All claims expressed in this article are solely those of the authors and do not necessarily represent those of their affiliated organizations, or those of the publisher, the editors and the reviewers. Any product that may be evaluated in this article, or claim that may be made by its manufacturer, is not guaranteed or endorsed by the publisher.
